# *Nocardia* brain abscess in a patient with diabetes: a case report

**DOI:** 10.1186/s13256-023-04071-0

**Published:** 2023-08-09

**Authors:** Noelle Boctor, Paul Aronowitz

**Affiliations:** 1 Department of Internal Medicine, University of California, Davis, 2315 Stockton Blvd, Suite 2P101, Sacramento, CA 95817 USA; 2 University of California, Davis, 4150 V St, PSSB 3100, Sacramento, CA 95817 USA

**Keywords:** *Nocardia*, Brain abscess, Diabetes, Immunocompromised

## Abstract

**Background:**

*Nocardia* are aerobic Gram-positive bacilli that can invade multiple organ systems, including the brain and lungs. It is most frequently found in patients who are immunocompromised. Invasive nocardial disease is a potentially life-threatening infection that can pose a diagnostic challenge.

**Case presentation:**

Our case details a 76-year-old Indian woman with poorly-controlled diabetes mellitus admitted for confusion and falls. Imaging revealed intracranial abscesses and necrotic masses in the mediastinum and lungs. The suspected diagnosis was tuberculosis; however, she underwent extensive workup without a final diagnosis. Ultimately, a craniotomy with partial brain abscess resection was performed. Dura matter samples revealed *Nocardia farcinica*.

**Conclusions:**

This case illustrates the importance of considering *Nocardia* in patients with brain abscesses, particularly in those with immunocompromised states and demonstrates the diagnostic challenges that may arise in definitively making this diagnosis. Invasive procedures may be needed for diagnostic confirmation.

## Background

*Nocardia* are aerobic partially acid-fast Gram-positive bacilli that can invade multiple organ systems, including the brain, lung, and skin. This infection is most commonly found in immunocompromised patients. *Nocardia* brain abscesses are uncommon, only accounting for 2% of all brain abscesses [1, 2] and therefore may not be considered in the initial differential diagnosis for patients presenting with brain abscess. However, its mortality rate has been reported to be up to 31% compared with other infectious brain abscesses (< 10%) [3], demonstrating how crucial it is to obtain a timely and accurate diagnosis. We discuss a case of an elderly patient with poorly-controlled diabetes presenting with neurologic symptoms found to have intracranial brain abscesses that were initially suspected to be due to tuberculosis, but ultimately found to be due to *Nocardia*. This case demonstrates the diagnostic challenges that clinicians may face in definitively diagnosing this insidious disease. Given the high mortality, it further emphasizes the importance of considering *Nocardia* in immunocompromised patients with brain abscess and that invasive procedures may be required to make the diagnosis.

## Case presentation

A 76-year-old Indian woman with a long-standing history of poorly controlled diabetes mellitus and dementia was visiting her family in the USA from India, when she presented from an external hospital with worsening confusion over a period of 4 weeks and falls toward her left side over a period of 1 week. At baseline, the patient was forgetful and confused, with intermittent visual hallucinations, but was able to carry a conversation. On presentation, her family reported she had an inability to carry a conversation, more frequent hallucinations, and new falls to the left side. Review of systems was notable for a dry cough, which had started 1 month prior to presentation, after arrival to the USA.

**Fig. 1:**
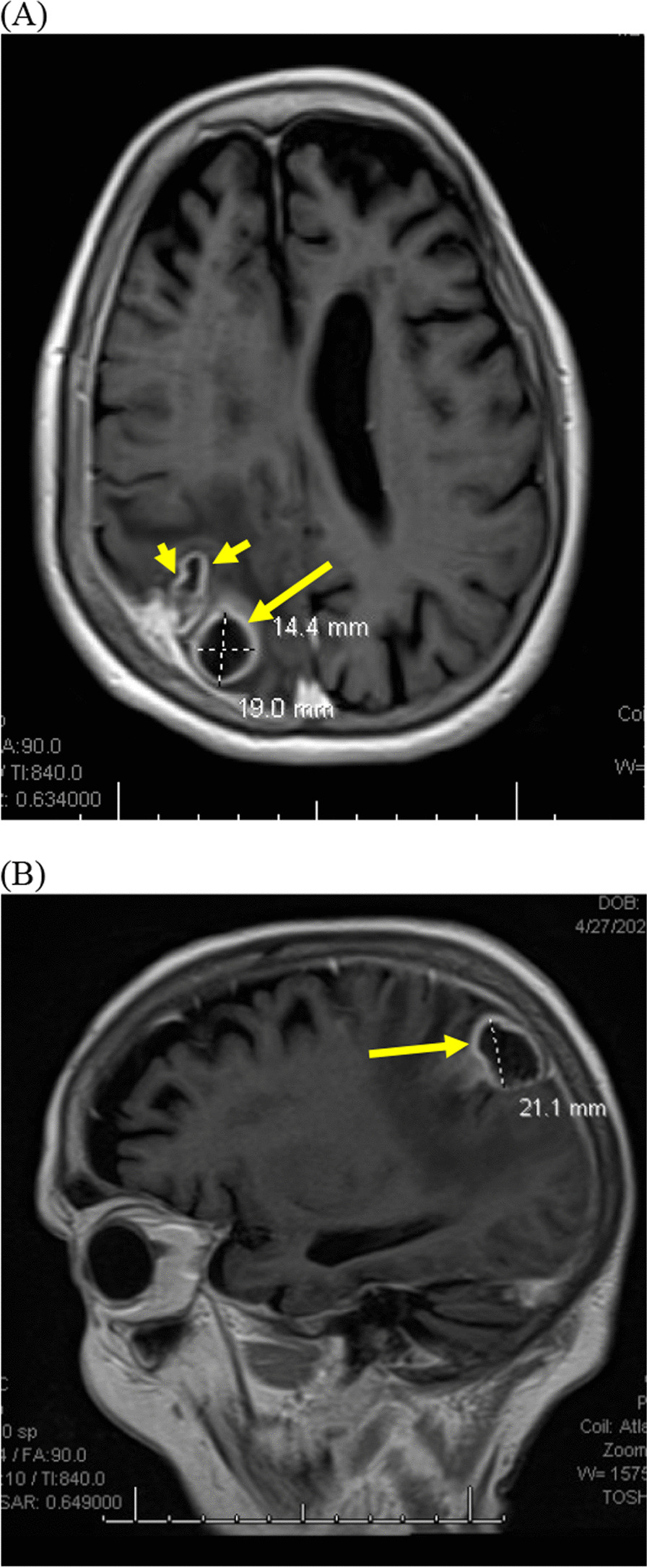
Brain magnetic resonance imaging images in transverse (**A**) and sagittal (**B**) views demonstrate multiple enhancing lesions (short yellow arrows) with vasogenic edema in the right parietal lobe consistent with abscesses, with the largest abscess measuring 1.4 cm × 1.9 cm × 2.1 cm (long yellow arrow)

Physical examination was notable for orientation only to self, which her family reported was changed from baseline. She had left-sided neglect, 5/5 strength in the right upper and lower extremities, 2/5 strength in the left upper extremity, and 3/5 strength in the left lower extremity. Otherwise, sensory exam and cranial nerves were intact. Her lungs were clear to auscultation bilaterally. Laboratory studies showed serum glucose of 268 mg/dL and white blood cell count (WBC) of 6200 cells/µL with 87% neutrophils. Hemoglobin A1c was 11.2%. Computed tomography (CT) of the head revealed a subacute right subdural hematoma and multiple right-sided intracranial ring-enhancing lesions. Magnetic resonance imaging (MRI) of the brain revealed multiple right parietal abscesses (Fig. 1).

She was started on intravenous antimicrobials including vancomycin, ceftriaxone, and metronidazole, as well as levetiracetam for seizure prevention. A lumbar puncture revealed a low opening pressure, glucose level of 79 mg/dL, protein of 105 mg/dL, and WBC of 225 cells/µL that was predominately neutrophilic. Due to concern for tuberculosis (TB), CT scan of the chest and thoracic and lumbar spine were obtained. Chest CT showed a necrotic mediastinal soft tissue mass involving the esophagus and multiple bilateral upper lobe pulmonary nodules. She was placed on airborne precautions for suspected active TB infection and collection of three sputum samples for TB smear, culture, and polymerase chain reaction (PCR) testing was attempted. She was unable to produce adequate sputum samples and therefore underwent bronchoscopy with lavage and transbronchial fine needle aspiration. Bacterial and fungal cultures, cytology, acid-fast bacilli (AFB) smears, and TB PCR testing failed to reveal the etiology of the pulmonary lesions. Pulmonary lymph node aspiration samples were negative for malignant cells. Blood cultures and serology for human immunodeficiency virus (HIV), *Histoplasma* antigen, cryptococcal antigen, and *Echinococcus* antibody were all unremarkable. Cerebrospinal fluid cultures were also unrevealing for the etiology of her brain abscesses. Consulting neurosurgeons then performed a right parietal craniotomy and partial resection of the largest abscess. Due to continued high suspicion for disseminated TB, she was empirically started on treatment with rifampin, isoniazid, pyrazinamide, and ethambutol immediately after surgery. AFB smear and *Mycobacterium tuberculosis* polymerase chain reaction testing from the brain abscess were all negative for TB. However, one of four dura mater tissue samples obtained during surgery subsequently grew *Nocardia farcinica*. Further tissue PCR testing at an external institution also revealed *Nocardia farcinica*.

Tuberculosis therapy was stopped after she had received it for five days, and based on our institution’s *Nocardia* susceptibility patterns, the patient was started on intravenous linezolid, trimethoprim-sulfamethoxazole, and meropenem. Seven days later and 3 weeks after presentation to our hospital, she was transferred back to the hospital she first presented at to be closer to her family, receiving oral linezolid, oral trimethoprim-sulfamethoxazole, and intravenous imipenem with recommendations to her treating physicians of follow-up susceptibilities of *Nocardia* to guide further treatment. Repeat MRI brain and a CT chest scans in 2 weeks, as well as antimicrobial therapy for at least 12 months, were recommended. On discharge from our hospital, she remained confused, but her agitation had improved, and she no longer exhibited weakness in the left upper and lower extremities. Susceptibilities of *Nocardia farcinica* resulted two days after her transfer. Results were communicated to the external hospital and she was switched to amikacin, levofloxacin, and linezolid. One month later, she presented to an external hospital for abdominal pain and was found to be suffering a myocardial infarction. CT scan of the head at the time showed resolution of posterior superior right parietal lobe abscesses with near complete resolution of associated vasogenic edema. Her family opted to transition her to comfort care and she died 3 days later.

## Discussion and conclusions

*Nocardia* are aerobic partially acid-fast Gram-positive bacilli that can invade multiple organs including lung, skin, and the central nervous system (CNS). The most common portal of entry is via inhalation or skin breakdown, and it is often found in soil, water, and vegetable matter [4]. It is usually diagnosed in immunocompromised patients, especially those with decreased cell-mediated immunity [5]. Our patient had poorly controlled diabetes mellitus, which has been shown to increase susceptibility to *Nocardia* infection [3, 5]. Several case reports have described nocardial brain abscesses in immunocompromised patients, citing up to 71% of patients with CNS nocardiosis as more likely to be immunocompromised, and that between 29% and 36% of patients with *Nocardia* brain abscess have diabetes [6, 7]. CNS imaging should be considered in immunocompromised patients with known nocardiosis, especially in the setting of neurologic symptoms [5], such as in our patient who presented with altered mentation from baseline and left-sided weakness. Due to its frequent pulmonary mode of entry, other common differential diagnoses are tuberculosis, mycoses, neoplasm, or other bacterial lung abscesses [4].

*Nocardia* infection of the CNS leading to brain abscess can occur due to hematogenous spread and has substantial mortality of up to 55% in immunocompromised, and as high as 20% in immunocompetent patients [1, 5]. *Nocardia* brain abscess can develop over months to years and can be insidious [4]. Extraneural *Nocardia* is more prevalent (71%) than CNS nocardiosis, and thus in disseminated *Nocardia* brain imaging is critical to rule out intracranial involvement [1].

Definitive diagnosis of *Nocardia* brain abscess is confirmed with cerebrospinal or brain aspirate cultures, thus making the diagnosis is often delayed due to the bacteria’s slow-growing nature [8]. Surgical excision or aspiration of the abscess may be indicated if it measures greater than 2.5 cm [9]. It has been suggested that unlike other bacterial abscesses, craniotomy and excision of the entire abscess and wall is usually more effective than aspiration and drainage [1, 9]. Smears usually show weakly acid-fast staining Gram positive branching filaments. Specimens with rapidly growing mixed flora can mask *Nocardia* species from growing, so the specimens often have a higher yield if mixed with antibiotics, adding to its microbiologic diagnostic challenges [3]. Other molecular methods have also been suggested for ease of diagnosis, but these are not yet widely used in clinical laboratories and are often limited to research-oriented laboratories [3].

Standard treatment for *Nocardia* brain abscess is a sulfonamide, such as trimethoprim-sulfamethoxazole (TMP-SMX), as these agents have good cerebrospinal fluid penetration [1, 4, 5]. Carbapenems, tetracyclines, fluoroquinolones, linezolid, and some cephalosporins have also been shown to be active against *Nocardia* species [5]. Case reports have shown successful treatment with these agents in patients with *Nocardia* brain abscess [3, 10]. Combination therapy with two susceptible antibiotics has also been shown to provide enhanced treatment efficacy. For most forms of nocardiosis, initial combination drug therapy, and in severe cases triple drug therapy, is now recommended [5]. In patients with CNS involvement, it is imperative to use antimicrobial agents with CNS penetration, such as TMP-SMX and cephalosporins. Some case reports have found that occasionally, surgical management of *Nocardia* brain abscess in conjunction with combination antibiotics is needed for successful treatment [9, 11]. Treatment is usually for a prolonged duration to prevent relapse and should be continued until the patient demonstrates clinical improvement and microbial drug susceptibilities are obtained. In immunocompromised patients with CNS disease, treatment duration with antimicrobial therapy last at least 12 months, while patients who are immunocompetent with non-CNS nocardiosis can be treated for 6–12 months [5].

*Nocardia* are bacterial pathogens with high mortality that require an elevated index of suspicion in immunocompromised patients presenting with brain abscesses, such as our patient. Although *Nocardia* infection can result in serious invasive disease, complete treatment is possible and hinges on early diagnosis and treatment initiation to minimize mortality.

## Data Availability

Not applicable.

## Abbreviations

WBC: White blood cell count
CT: Computed tomography
MRI: Magnetic resonance imaging
TB: Tuberculosis
PCR: Polymerase chain reaction
AFB: Acid-fast bacilli
CNS: Central nervous system
TMP-SMX: Trimethoprim-sulfamethoxazole
